# Tropical Sprue: A Rare Cause of Malabsorption Syndrome

**DOI:** 10.7759/cureus.53748

**Published:** 2024-02-06

**Authors:** Catarina Costa, Francisca Bartilotti Matos, Diogo Carvalho Sá, João Neves Maia

**Affiliations:** 1 Department of Internal Medicine, Centro Hospitalar Universitário de Santo António, Porto, PRT; 2 Department of Infectious Diseases, Centro Hospitalar de Vila Nova de Gaia/Espinho, Vila Nova de Gaia, PRT; 3 Department of Pathology, Centro Hospitalar Universitário de Santo António, Porto, PRT

**Keywords:** small intestinal bacterial overgrowth, vitamin b12 deficiency, malabsorption syndrome, chronic diarrhea, tropical sprue

## Abstract

Chronic diarrhea is a common disorder in tropical regions, affecting residents, visitors, and even expatriates. It may stem from a myriad of infectious, inflammatory, and even malignant causes. In patients in whom no etiology has been found, tropical sprue (TS) is an important diagnosis to consider. We report the case of a 60-year-old man originally from Guatemala, presenting with chronic diarrhea and megaloblastic anemia due to severe vitamin B12 deficiency. Biopsies of the small bowel revealed partial villous atrophy and inflammatory infiltrate with the participation of eosinophils. The diagnosis of TS was established after exclusion of other causes of malabsorption syndrome. This is a disease of unknown etiology with complex and multifactorial pathophysiology, with an important component of intestinal dysbiosis. Antibiotics and vitamin supplementation are the pillars of therapy. Awareness of this disorder is essential in preventing delayed diagnosis and subsequent morbidity.

## Introduction

Tropical sprue (TS) is a rare and acquired cause of malabsorption syndrome, endemic to tropical regions like Southeast Asia (notably India) and Central America [1,2]. Its etiology is unknown and is characterized by chronic diarrhea, multiple nutritional deficiencies, and mucosal abnormalities in the small bowel [3]. The incidence of this disease has decreased dramatically over the past few decades, which has been attributed to improvements in sanitation, greater food and water hygiene, and prevalent use of antibiotics [1,3]. We present a case of sporadic TS, made all the more relevant in the current context of globalization with facilitated movement of people from endemic to non-endemic regions.

## Case presentation

A 60-year-old man presented to the emergency department with a six-month history of steatorrhea, asthenia, anorexia, nausea, and significant unintentional weight loss (25% of usual body weight). He also reported occasional episodes of small painful mouth ulcers. The patient was originally from Guatemala and had immigrated to Portugal two months before presentation. He lived in an urban area with adequate sanitation in both countries, only consumed bottled water, and denied contact with animals, recent antibiotic use, and consumption of unpasteurized or raw foods.

At presentation, the patient was emaciated but had no signs of acute distress or any other physical examination abnormalities. Laboratory tests were remarkable for macrocytic anemia, marked anisocytosis and hypersegmented neutrophils on the peripheral blood smear, vitamin D and E deficiency, and levels of vitamin B12 that were below the threshold of detection. There was no peripheral eosinophilia and a human immunodeficiency virus serology was negative. These and other laboratory data can be found in Table 1.

**Table 1:** Laboratory data on admission

Variable	On Admission	Reference Range
White-cell count (/µL)	6840	4000-11000
Hemoglobin (g/dL)	8.9	13-17
Mean Corpuscular Volume (fL)	111.8	83-101
Platelets (/µL)	142000	150000-40000
Erythrocyte Sedimentation Rate (mm)	45	0-12
C Reactive Protein (mg/L)	<0.6	0-5
Albumin (g/dL)	4.65	3.5-5
Vitamin B12 (pg/mL)	<100	191-663
Folic Acid (ng/mL)	3.9	3.9-26.8
Vitamin A (µmol/L)	1.36	1.05-2.45
Vitamin D (nmol/L)	29	50-150
Vitamin E (µmol/L)	10.2	12-42
Ferritin (ng/mL)	484	12.8-454
Transferrin Saturation (%)	40	15-45
International Normalized Ratio	1.7	-

**Figure 1:**
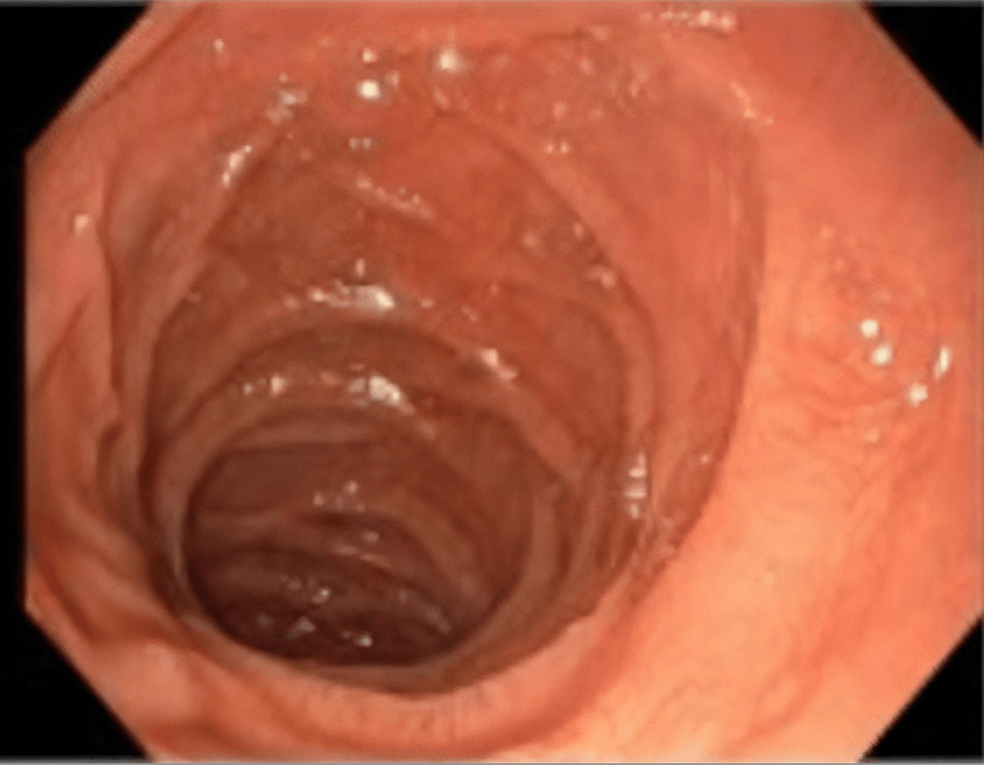
Endoscopic appearance of the second portion of the duodenum

These findings suggested a picture of malabsorption syndrome, so the patient was admitted for further testing. He was started on vitamin supplementation with symptom improvement. Computed tomography of the chest, abdomen, and pelvis was unrevealing. Antibodies for pernicious anemia and celiac disease were negative and stool elastase levels were normal. Microbiological examination of the stool was negative for ova and parasites and only found commensal flora. The patient underwent upper endoscopy and colonoscopy, which described a granite appearance in the duodenum (Figure 1), with no other morphologic changes.

**Figure 2:**
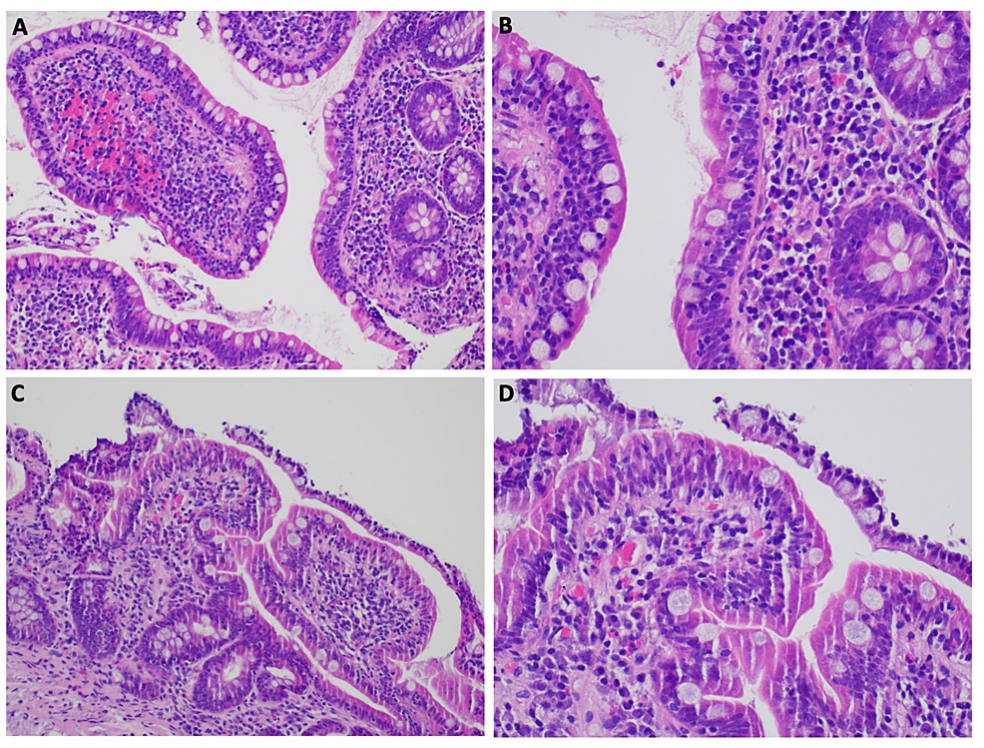
Ileum (A and B, H&E 200x and H&E 400x) and duodenum (C and D, H&E 200x and H&E 400x) biopsies showing mild villous blunting and an inflammatory infiltrate of lymphocytes, plasma cells, and eosinophils in the lamina propria. There are an increased number of intraepithelial lymphocytes

The pathology report of the biopsies done during the endoscopic studies eventually revealed villous atrophy, lymphoplasmacytic infiltrate of the lamina propria with participation of eosinophils and intraepithelial lymphocytosis in the duodenum and terminal ileum (Figure 2). A polymerase chain reaction test for Tropheryma whipplei in the duodenal tissue was negative.

After extensive exclusion of other causes of malabsorption syndrome, the combination of the prominent megaloblastic anemia, the histopathologic changes with negative microbiology tests, and the epidemiological context were consistent with a diagnosis of TS. The patient was started on a three-month course of doxycycline with complete clinical remission, resolution of the anemia, and normalization of the vitamin deficits. 

## Discussion

TS is a disorder of elusive etiology. While there is evidence of a probable infectious cause behind the development of TS, extensive research has not been able to consistently link a specific microbiological agent to the disease [4,5]. Regardless, intestinal dysbiosis seems to play a central role in the pathophysiology of TS [4,5]. This mechanism is traduced by bacterial colonization and overgrowth [5], enterotoxin production [1], neuro-hormonal dysregulation, and an abnormal host immune response causing chronic inflammation [5]. The resulting enterocyte damage triggers malabsorption of various nutrients, especially carbohydrates, lipids, lipid-soluble proteins, folic acid, and vitamin B12 [5], as highlighted by this case report. The increase of intra-luminal fat in the small bowel leads to a prolongation of the gut transit time, which contributes to intestinal stasis and perpetuation of bacterial overgrowth.

This explains the clinical manifestations of TS, which include chronic diarrhea (often steatorrhea), bloating and abdominal pain, anorexia, and weight loss [1]. Fever is uncommon [4]. Other manifestations will vary depending on the vitamin deficiencies at play. In this case, megaloblastic anemia due to vitamin B12 deficiency was one of the key features.

The main pathological small bowel changes are partial villous atrophy, increased crypt depth, and increased inflammatory cell population of the lamina propria that may include eosinophils and elevated intraepithelial lymphocyte counts [1]. This eosinophil-rich infiltrate is less commonly reported but was also found in this case. 

The challenge of diagnosing TS resides in its clinical similarities when compared to other malabsorption syndromes, namely celiac disease or specific infectious causes [4]. It is therefore unsurprising that TS is a diagnosis of exclusion that requires rule out of a causal infectious agent.

While spontaneous recovery might be possible in mild forms of the disease, most patients will need pharmacological treatment to ensure full clinical resolution [1]. 

Given the presumed role of bacterial overgrowth in pathophysiology, the cornerstone of treatment is antibiotic therapy with tetracycline or doxycycline for 3 to 6 months, along with vitamin supplementation [1,3]. This patient achieved a complete clinical response, even though the histologic changes were not reevaluated. Prognosis is usually good in TS since recovery is usually complete and permanent in expatriates [1]. In patients that remain in endemic regions, relapses can occur in approximately 50% of cases [3].

## Conclusions

Clinicians in non-endemic areas should be alert to exotic causes of malabsorption syndrome, especially in patients with appropriate epidemiological context and after exclusion of more common disorders, since it leads to appropriate treatment and better prognosis.

## References

[1] Nath SK (2005). Tropical sprue. Curr Gastroenterol Rep.

[2] Westergaard H (2004). Tropical sprue. Curr Treat Options Gastroenterol.

[3] Ramakrishna BS, Venkataraman S, Mukhopadhya A (2006). Tropical malabsorption. Postgrad Med J.

[4] Lim ML (2001). A perspective on tropical sprue. Curr Gastroenterol Rep.

[5] Ghoshal UC, Srivastava D, Verma A, Ghoshal U (2014). Tropical sprue in 2014: the new face of an old disease. Curr Gastroenterol Rep.

